# Fibromyalgia in migraine: a retrospective cohort study

**DOI:** 10.1186/s10194-018-0892-9

**Published:** 2018-07-31

**Authors:** Mark Whealy, Sanjeev Nanda, Ann Vincent, Jay Mandrekar, F. Michael Cutrer

**Affiliations:** 1 Department of Neurology, Division of Headache, Mayo Clinic Rochester, 200 First Street SW, Rochester, MN 55905 USA; 2 Department of Medicine, Mayo Clinic Rochester, 200 First Street SW, Rochester, MN 55905 USA; 3 Department of Health Sciences Research, Mayo Clinic Rochester, 200 First Street SW, Rochester, MN 55905 USA

**Keywords:** Fibromyalgia, Migraine, MIDAS, PHQ-9, Headache

## Abstract

**Background:**

Migraine is a common and disabling disorder. Fibromyalgia has been shown to be commonly comorbid in patients with migraine and can intensify disability. The aim of this study was to determine if patients with co-morbid fibromyalgia and migraine report more depressive symptoms, have more headache related disability, or report higher intensity of headache as compared to patients with migraine only. Cases of comorbid fibromyalgia and migraine were identified using a prospectively maintained headache database at Mayo Clinic Rochester. One-hundred and fifty seven cases and 471 controls were identified using this database and the Mayo Clinic electronic medical record.

**Findings:**

Depressive symptoms as assessed by PHQ-9, intensity of headache, and migraine related disability as assessed by MIDAS were primary measures used to compare migraine patients with comorbid fibromyalgia versus those without. Patients with comorbid fibromyalgia reported significantly higher PHQ-9 scores (OR 1.08, *p* < .0001) and headache intensity scores (OR 1.149, *p* = .007). There was no significant difference in migraine related disability (OR 1.002, *p* = .075). Patients with fibromyalgia were more likely to score in a higher category of depression severity (OR 1.467, *p* < .0001) and more likely to score in a higher category of migraine related disability (OR 1.23, *p* = .004).

**Conclusion:**

Patients with comorbid fibromyalgia and migraine report more depressive symptoms, higher headache intensity, and are more likely to have severe headache related disability as compared to controls without fibromyalgia. Clinicians who care for patients with migraine may consider screening for comorbid fibromyalgia particularly in patients with moderate to severe depressive symptoms, high headache intensity and/or high headache related disability. This is the first matched study to look at these characterisitcs, and it replicates previous findings from unmatched studies.

## Introduction

Fibromyalgia is a chronic pain disorder characterized by chronic widespread pain, debilitating fatigue, headache, cognitive difficulties and mood disorders and is reported to be more prevalent in females [1]. Several studies have reported comorbid fibromyalgia in patients with migraine, ranging from 18% to 35.6% [1–6]. It is known that central sensitization plays a role in fibromyalgia and chronic migraine [7]. Fibromyalgia and migraine are debilitating pain disorders and thus can add to the morbidity of the other disorder if present together and can significantly impact the quality of life in patients who have both disorders [1, 2].

The aim of this study was to determine if patients with comorbid fibromyalgia and migraine differed in their reports of depressive symptoms, headache intensity and/or migraine related disability compared to age and sex matched controls with migraine only. This is the first matched study to look at these characteristics with the idea to replicate previous findings.

## Methods

### Subjects

One hundred and fifty seven cases of comorbid migraine and fibromyalgia and 471 control subjects (three age and sex matched controls per patient) with migraine and no fibromyalgia were identified using a prospectively collected and maintained headache database [8]. The Mayo Clinic electronic medical record was then used to confirm or deny the presence of the diagnosis of fibromyalgia. Patients in this database were identified based on information provided on their initial visit information; with initial visits between 2012 and 2017. All patients were evaluated in the Headache Clinic at Mayo Clinic in Rochester by UCNS board certified headache specialists to verify questionnaire data. Data on MIDAS, PHQ-9 and headache intensity was extracted from the database. This study was approved by the Mayo Clinic Institutional Review Board.

### Eligibility criteria

Eligibility criteria of cases included a diagnosis of any subtype of migraine in addition to a diagnosis of fibromyalgia. Eligibility criteria for controls included a diagnosis of any subtype of migraine without a diagnosis of fibromyalgia. The International Classification of Headache Disorders, 3rd edition (beta version), diagnostic criteria for migraine were used [9]. The 2010 American College of Rheumatology diagnostic criteria for fibromyalgia were used [10].

### Migraine disability assessment scale (MIDAS) [11]

The MIDAS is a validated and reliable scale to help determine a subject’s migraine related disability over the prior 3 months. It asks about days that a subject missed or had reduced production in 3 domains (school/work, household work, and social/leisure/family activities). Scores are as follows: Grade I: 0–5 little or no disability, Grade II: 6–10 mild disability, Grade III: 11–20 moderate disability, Grade IV: ≥ 21 severe disability.

### PHQ-9 [12]

The PHQ-9 is a validated questionnaire used to screen for depression. There are 9 statements of depressive symptoms which are rated from 0 to 3 (not at all/several days/more than half the days/nearly every day) over the previous 2 weeks. Scores are as follows: 0–4 none, 5–9 mild, 10–14 moderate, 15–19 moderately severe, 20–27 severe.

### Statistical analyses

Descriptive statistics (mean, standard deviation (SD), and percent) were used to characterize the sample. Conditional logistic regression was used to compare the cases and controls. Analyses were conducted with SAS Statistical Software (Version 9.4, SAS Institute Inc., Cary, NC, USA). *P* values < .05 were considered significant.

## Findings

A total of 157 cases of comorbid fibromyalgia and migraine were identified between 2012 and 2017 for inclusion in the study. Three age and sex matched controls with migraine only were identified for each case for a total of 471 control subjects. BMI, number of headache days in the 4 weeks preceding the initial visit, age of onset of headaches, number of subjects with 15 or more headache days in the last 4 weeks, and average duration of migraines are shown in Table 1. There was no difference between the cases and controls with regards to any of the above characteristics.

**Table 1:** BMI, migraine duration, age of headache onset, number of headache days last 4 weeks, and number of subjects with 15 or more headache days is last 4 weeks. OR- odds ratio, 95% CI, 95% confidence interval

	Cases (*n* = 157)	Controls (*n* = 471)	OR	95% CI	*p*-value
BMI, mean (SD)	28.5 (6.7)	27.8 (6.9)	1.02	0.99 to 1.04	0.24
Duration of migraine attacks (hrs), mean (SD)	29.6 (35.5)	30.8 (95.1)	1.0	0.997 to 1.002	0.88
Age of onset of headaches (years), mean (SD)	20.6 (13.5)	21.1 (13.0)	0.997	0.98 to 1.01	0.67
Headache days in last 4 weeks, mean (SD)	18.7 (8.8)	18.3 (9.1)	1.01	0.99 to 1.03	0.63
15 or more headache days in last 4 weeks, n (%)	111 (70.7)	311 (66.0)	1.24	0.84 to 1.83	0.28

The cases reported higher PHQ-9 scores and were more likely to score in a higher severity category on the PHQ-9. Average intensity of headaches rated on a 10 point scale (0-no pain; 10-worst pain imaginable) was higher in cases as compared to controls. Although there was no significant difference in the total MIDAS score between cases and controls, a significantly higher proportion of cases scored a higher grade of migraine related disability on the MIDAS (Table 2).

**Table 2:** Comparing MIDAS, PHQ-9 and average headache intensity between cases and controls

	OR	95% CI	*p*-value
Headache Intensity	1.15	1.04 to 1.27	0.0065
MIDAS	1.002	1 to 1.004	0.0747
MIDAS (categorical)	1.23	1.01 to 1.50	0.0374
Grade I: 0–5			
Grade II: 6–10			
Grade III: 11–20			
Grade IV: > 20			
PHQ-9	1.08	1.04 to 1.11	<.0001
PHQ-9 (categorical)	1.47	1.24 to 1.74	<.0001
0–4: Minimal to none			
5–9: Mild			
10–14: Moderate			
15–19: Moderately severe			
> 19: Severe			

## Conclusion

Fibromyalgia and migraine are commonly co-morbid disorders. There is a high prevalence of fibromyalgia in migraineurs [1–6] and a high prevalence of migraine and other headaches in patients with fibromyalgia [13–15]. Scores on PHQ-9 and average headache intensity were significantly higher in the patients with comorbid fibromyalgia. Cases were more likely to score in a higher severity category for both the MIDAS and the PHQ-9. This study confirms previous reports of increased headache related disability [2], depression [1, 3], and headache severity [2] in patients with comorbid fibromyalgia and migraine as compared to those with migraine only.

The limitations of this study include the fact that the majority of the patients had chronic migraine which is associated with medication overuse and medication overuse was not accounted for in this paper.

The presence of fibromyalgia has been correlated with lower quality of life [1, 2] in patients with migraine, making it important to know when to screen for symptoms of fibromyalgia in the migraine population. Our findings suggest that it is important to inquire about comorbid fibromyalgia as this needs to be taken into consideration with regards to creating an optimal individualized treatment plan. On the basis of this study, it would be reasonable to screen for symptoms fibromyalgia in a migraine patient when they report a number of depressive symptoms, severe headache intensity, or severe headache related disability.

## Abbreviations

BMI: Body mass index
CI: Confidence interval
MIDAS: Migraine disability assessment score
OR: Odds ratio
PHQ-9: Patient health questionnaire 9
UCNS: United Council for Neurologic Subspecialties
